# Investigating the factors underlying adaptive functioning in autism in the EU‐AIMS Longitudinal European Autism Project

**DOI:** 10.1002/aur.2081

**Published:** 2019-02-11

**Authors:** Julian Tillmann, Antonia San José Cáceres, Chris H. Chatham, Daisy Crawley, Rosemary Holt, Bethany Oakley, Tobias Banaschewski, Simon Baron‐Cohen, Sven Bölte, Jan K. Buitelaar, Sarah Durston, Lindsay Ham, Eva Loth, Emily Simonoff, Will Spooren, Declan G. Murphy, Tony Charman, the EU‐AIMS LEAP group, Jumana Ahmad, Sara Ambrosino, Bonnie Auyeung, Sarah Baumeister, Christian Beckmann, Thomas Bourgeron, Carsten Bours, Michael Brammer, Daniel Brandeis, Claudia Brogna, Yvette de Bruijn, Bhismadev Chakrabarti, Ineke Cornelissen, Flavio Dell’ Acqua, Guillaume Dumas, Christine Ecker, Jessica Faulkner, Vincent Frouin, Pilar Garcés, David Goyard, Hannah Hayward, Joerg Hipp, Mark H. Johnson, Emily J.H. Jones, Prantik Kundu, Meng‐Chuan Lai, Xavier Liogier D'ardhuy, Michael Lombardo, David J. Lythgoe, René Mandl, Luke Mason, Andreas Meyer‐Lindenberg, Carolin Moessnang, Nico Mueller, Laurence O'Dwyer, Marianne Oldehinkel, Bob Oranje, Gahan Pandina, Antonio M. Persico, Barbara Ruggeri, Amber Ruigrok, Jessica Sabet, Roberto Sacco, Roberto Toro, Heike Tost, Jack Waldman, Steve C.R. Williams, Caroline Wooldridge, Marcel P. Zwiers

**Affiliations:** ^1^ Department of Psychology, Institute of Psychiatry, Psychology & Neuroscience King's College London London UK; ^2^ Department of Applied Psychology: Health, Development, Enhancement, and Intervention University of Vienna Vienna Austria; ^3^ Department of Forensic and Neurodevelopmental Sciences, Institute of Psychiatry, Psychology and Neuroscience King's College London London UK; ^4^ F. Hoffmann La Roche Innovation Center Basel Basel Switzerland; ^5^ Autism Research Centre, Department of Psychiatry University of Cambridge Cambridge UK; ^6^ Child and Adolescent Psychiatry, Central Institute of Mental Health, Medical Faculty Mannheim University of Heidelberg Mannheim Germany; ^7^ Center for Neurodevelopmental Disorders (KIND), Division of Neuropsychiatry, Department of Women's and Children's Health Karolinska Institutet Stockholm Sweden; ^8^ Child and Adolescent Psychiatry, Center of Psychiatry Research Stockholm County Council Stockholm Sweden; ^9^ Radboud University Nijmegen Medical Center Donders Institute for Brain, Cognition and Behaviour, Department of Cognitive Neuroscience Nijmegen The Netherlands; ^10^ Karakter Child and Adolescent Psychiatry University Center Nijmegen The Netherlands; ^11^ Department of Psychiatry, Brain Center Rudolf Magnus University Medical Center Utrecht Utrecht The Netherlands; ^12^ Sackler Institute for Translational Neurodevelopment, Institute of Psychiatry, Psychology and Neuroscience King's College London London UK; ^13^ Department of Child and Adolescent Psychiatry, Institute of Psychology, Psychiatry and Neuroscience King's College London London UK; ^14^ South London and Maudsley NHS Foundation Trust (SLaM) London UK

**Keywords:** autism spectrum disorder, adaptive functioning, intellectual functioning, symptom severity, psychiatric symptoms

## Abstract

Individuals with autism spectrum disorder (ASD) exhibit significant impairments in adaptive functioning that impact on their ability to meet the demands of everyday life. A recurrent finding is that there is a pronounced discrepancy between level of cognitive ability and adaptive functioning, and this is particularly prominent among higher‐ability individuals. However, the key clinical and demographic associations of these discrepancies remain unclear. This study included a sample of 417 children, adolescents, and adults with ASD as part of the EU‐AIMS LEAP cohort. We examined how age, sex, IQ, levels of ASD symptom and autistic trait severity and psychiatric symptomatology are associated with adaptive functioning as measured by the Vineland Adaptive Behavior Scales‐Second Edition and IQ‐adaptive functioning discrepancies. Older age, lower IQ and higher social‐communication symptoms were associated with lower adaptive functioning. Results also demonstrate that older age, higher IQ and higher social‐communication symptoms are associated with greater IQ‐adaptive functioning discrepancy scores. By contrast, sensory ASD symptoms, repetitive and restricted behaviors, as well as symptoms of attention deficit/hyperactivity disorder (ADHD), anxiety and depression, were not associated with adaptive functioning or IQ‐adaptive functioning discrepancy scores. These findings suggest that it is the core social communication problems that define ASD that contribute to adaptive function impairments that people with ASD experience. They show for the first time that sensory symptoms, repetitive behavior and associated psychiatric symptoms do not independently contribute to adaptive function impairments. Individuals with ASD require supportive interventions across the lifespan that take account of social‐communicative ASD symptom severity. ***Autism Res** 2019, 12: 645–657*. © 2019 The Authors. *Autism Research published by International Society for Autism Research* published by Wiley Periodicals, Inc.

**Lay summary:**

This study investigated key clinical and demographic associations of adaptive functioning impairments in individuals with autism. We found that older age, lower IQ and more severe social‐communicative symptoms, but not sensory or repetitive symptoms or co‐occurring psychiatric symptoms, are associated with lower adaptive functioning and greater ability‐adaptive function discrepancies. This suggests that interventions targeting adaptive skills acquisition should be flexible in their timing and intensity across developmental periods, levels of cognitive ability and take account of social‐communicative ASD symptom severity.

## Introduction

The term “adaptive behavior” refers to general societal expectancies about everyday functioning. In autism spectrum disorder (ASD), the Vineland Adaptive Behavior Scales [VABS; Sparrow, Balla, & Cicchetti, 1984; VABS‐II; Sparrow, Cicchetti, & Balla, 2005] has often been used to assess adaptive functioning in socialization, communication, self‐care, and life skills for personal independence and community living, as well as motor skills in individuals aged up to 6 years. VABS domains assessed map well to the core ASD symptoms of socialization and communication, as well as relevant measures of activities of daily living.

In ASD there is wide heterogeneity in the level of functioning across these domains, which is partly linked to development and level of cognitive abilities [Szatmari et al., 2015; Bal, Kim, Cheong, & Lord, 2015; Chatham et al., 2018; Farmer, Swineford, Swedo, & Thurm, 2018]. Although higher intellectual functioning is typically associated with better adaptive functioning, adaptive behavior tends to be more impaired than what would be expected based on general intellectual and cognitive ability [Bölte & Poustka, 2002; Klin et al., 2007; Charman et al., 2011; Kanne et al., 2011; Mouga, Almeida, Café, Duque, & Oliveira, 2015; Chatham et al., 2018]. The discrepancy between level of adaptive behavior and general intellectual level is particularly pronounced in individuals with ASD who have average or above average levels of cognitive functioning, where adaptive behavior has been found to lag one to two standard deviations behind IQ [Klin et al., 2007; Charman et al., 2011; Kanne et al., 2011; Kraper, Kenworthy, Popal, Martin, & Wallace, 2017]. In contrast, individuals with ASD and concurrent intellectual disabilities are more likely to exhibit adaptive behavior skills that are on par with or above their intellectual level [Bölte & Poustka, 2002; Perry, Flanagan, Geier, & Freeman, 2009]. Cross‐sectional and longitudinal studies also indicate that this discrepancy widens with age [Szatmari et al., 2009; Smith, Maenner, & Seltzer, 2012; Bal et al., 2015], suggesting that individuals with ASD are not acquiring adaptive skills at the same rate as their typically developing peers [Klin et al., 2007; Mouga et al., 2015]. Thus, despite having the necessary verbal and nonverbal processing skills, many individuals with higher intellectual abilities have difficulty translating their cognitive potential into functional independence. Adaptive functioning has been shown to be an important determinant of outcome in individuals with ASD [Farley et al., 2009], including educational attainment [De Bildt, Sytema, Kraijer, Sparrow, & Minderaa, 2005] and the level of independence that an individual can achieve in adulthood [Paul et al., 2004]. Identifying the factors that impede adaptive skill acquisition is therefore of great importance in planning more effective interventions to improve long‐term outcomes. Yet, beyond age and IQ, relatively little is known about the role of ASD core and associated psychiatric symptom severity in the magnitude of adaptive‐ability discrepancies. Identifying which specific aspects of the ASD phenotype, that is, social‐communicative symptoms, repetitive and restricted behaviors, or sensory symptoms, and how commonly associated psychiatric symptoms (e.g., ADHD, anxiety, and depression) are associated with adaptive functioning, could provide novel insight into unique contributions to variability in adaptive functioning and inform specific intervention programmes.

The few existing studies addressing this issue have however produced inconclusive results. Ashwood et al. [2015] found greater discrepancies between IQ and adaptive behavior, particularly in relation to social adaptive skills, in children with ASD and comorbid ADHD compared to an ADHD‐only group when controlling for age. Kraper et al. [2017] in a sample of cognitively able adults with ASD found that greater IQ‐adaptive discrepancies were associated to a small‐to‐moderate degree with more severe symptoms of depression and anxiety and social‐communicative symptoms characteristic of the ASD phenotype, but not ADHD‐related symptoms. Duncan and Bishop [2015] observed that the presence versus absence of a daily living skills deficit in relation to IQ in adolescents with ASD was only significantly related to older age and higher level of current social‐communicative symptoms on the Autism Diagnostic Interview—Revised [ADI‐R; Rutter, Couteur, & Lord, 2003]. Other predictor variables, including IQ, parent‐reported restrictive and repetitive behaviors (RRB), overall clinician‐rated ASD symptoms as measured by the Autism Diagnostic Observation Schedule [ADOS‐2; Lord et al., 2012], and internalizing and externalizing problem behaviors, were not associated with a higher likelihood of exhibiting a deficit. In addition to the relative lack of research in this area, methodological differences between studies further limit the generalisability of these findings, including small sample sizes, and limited age and IQ ranges studied. Further to this, only a few studies have simultaneously entered potential associated variables in regression models to contrast independent effects of specific aspects of the ASD phenotype and symptoms of associated psychiatric conditions.

### *The Current Study*

To overcome some of these previous limitations, we tested unique predictors of adaptive functioning as measured by the VABS and the discrepancy between IQ and adaptive functioning in a well‐characterized sample of individuals with ASD as part of the EU‐AIMS Longitudinal European Autism Project (LEAP) cohort [Charman et al., 2017; Loth et al., 2017]. LEAP includes participants across a broad age range from young children to adults and of different intellectual functioning. In this cohort we have comprehensive assessments not only of ASD symptoms using observational and questionnaire measures but also measures of common co‐occurring psychiatric symptoms: ADHD, anxiety and depression. Based on previous studies, we expected to find a larger IQ‐adaptive behavior discrepancy in older individuals and in individuals with higher IQs. Furthermore, we predicted that severity of core ASD symptoms would be associated with greater adaptive function impairments and IQ‐adaptive functioning discrepancies. We also tested whether co‐occurring psychiatric symptoms would be associated with greater adaptive function impairments and IQ‐adaptive functioning discrepancies above and beyond age and IQ and ASD symptoms. Previous studies have been lacking in the size of the samples studied (i.e., not “broad”) and the level of clinical characterization in relation to psychiatric symptoms (i.e., not ‘deep). In addition, few have tested potentially associated variables simultaneously in regression models. Given the scarcity of comprehensive studies, we did not generate a priori predictions in relation to the influence of co‐occuring psychiatric symptoms on adaptive behavior.

## Methods

### *Participants*

The sample consists of 417 participants with ASD ranging in age from 6 to 31 years (*M* = 16.9, *SD* = 5.95, *IQR* = 9.12) recruited as part of LEAP. Details of the study procedure, protocol, as well as inclusion/exclusion criteria and demographic and clinical characteristics of the cohort have been described elsewhere [Charman et al., 2017; Loth et al., 2017]. Descriptive statistics for the sample are listed in Table 1. Full‐Scale IQ (FSIQ) scores were available for 410 participants (98% of the sample) and ranged from 40 to 148, with a mean of 96.6 (*SD* = 20.27). At each site, an independent ethics committee approved the study. All participants (where appropriate) and/or their parent/legal guardian provided written informed consent.

**Table 1:** Sample Characteristics (*N* = 417)

	*N*	Mean	*SD*	Range
Sex (males: females)	301:116	–	–	–
Age in years	417	16.89	(5.95)	6–31
ADOS CSS‐SA	406	6.18	(2.65)	1–10
ADOS CSS‐RRB	406	4.92	(2.78)	1–10
SRS‐2	339	92.76	(30.38)	20–168
RBS‐R	337	16.82	(14.12)	0–90
SSP	240	137.73	(27.32)	53–190
ADHD—Inattentiveness	342	4.63	(3.18)	0–9
ADHD—Hyperactivity/Impulsivity	342	2.92	(2.90)	0–9
DAWBA—anxiety	346	2.55	(1.31)	0–5
DAWBA—depression	318	0.92	(1.24)	0–5
VABS Socialization	374	70.06	(16.44)	20–119
VABS Daily Living	373	72.40	(16.41)	25–131
VABS Communication	374	74.52	(17.22)	21–130
VABS ABC	371	70.27	(14.84)	20–121
Nonverbal IQ	410	97.48	(21.83)	44–150
Verbal IQ	406	95.66	(20.51)	41–160
Full‐scale IQ	410	96.61	(20.27)	40–148

*Note. SD*, standard deviation; ABCADOS CSS‐SA, RRB, autism diagnostic observation schedule calibrated severity scores for social affect and restricted and repetitive behaviors; SRS‐2, social responsiveness scale‐2 raw score; RBS‐R, repetitive behavior scale‐revised; SSP, short sensory profile; ADHD, DSM‐5 ADHD rating scale; DAWBA, development and well‐being assessment; VABS domain scores are standardized scores (age‐normalized: *M* = 100, *SD* = 15); VABS ABC, VABS Adaptive Behavior Composite; IQ, Intelligence Quotient.

### *Measures*

Adaptive functioning was measured using the VABS [VABS‐II; Sparrow et al., 2005]. The VABS is a semi‐structured parent interview that assesses adaptive functioning across three domains in >6‐year‐olds: communication, socialization, and daily living skills. For each domain, standard scores were obtained and combined to generate an Adaptive Behavior Composite (ABC) score. VABS standard scores have a mean of 100 (*SD* = 15), with lower scores indicating greater functional impairment.

General intellectual abilities were assessed using the *Wechsler Abbreviated Scales of Intelligence‐Second Edition* [WASI‐II; Wechsler, 2011], or if unavailable the *Wechsler Intelligence Scale for Children‐III/IV* [WISC‐III/IV; Wechsler, 1991, 2003] for children or *Wechsler Adult Intelligence Scale for Adults‐III/IV* [WAIS‐III/IV; Wechsler, 1997, 2008] for adults [see Charman et al., 2017 for a detailed description of IQ measures]. Standardized estimates of verbal IQ (VIQ), performance IQ (PIQ), and full‐scale IQ (FSIQ) were derived using IQ norms with *M* = 100 and *SD* = ±15.

The *Autism Diagnostic Observation Schedule* [ADOS‐G; Lord et al., 2000; ADOS‐2; Lord et al., 2012] is a semi‐structured, clinician‐administered instrument to evaluate aspects of social communication and interaction, stereotyped behaviors and restricted interests (see Supporting Information for additional information). ADOS‐2 algorithm totals can be used to derive a Calibrated Severity Score (CSS) for the core symptom domains of Social Communication (i.e., Social Affect), and Restricted and Repetitive Behaviors (RRB), as well as an overall indicator of ASD severity (CSS Total). The CSS ranges from 1 to 10, with higher scores indicating more severe ASD symptom severity.

The *Social Responsiveness Scale, Second Edition* [SRS‐2; Constantino & Gruber, 2012] is a quantitative measure of ASD traits and is composed of 65 items. Here we report parent‐report scores for total raw scores. The *Repetitive behavior scale‐revised* [RBS‐R; Bodfish, Symons, Parker, & Lewis, 2000], composed of 43 items, was used to derive parent‐reported total raw scores for restricted and repetitive behaviors relevant to the ASD phenotype, with higher scores indicating a greater level of atypical behaviors. Sensory processing atypicalities were assessed using the *short sensory profile* [SSP; Tomchek & Dunn, 2007] across 38 items, from which a total raw score was obtained (lower scores indicate more impairment) that reflect dysfunction across multiple sensory domains.

The *DSM‐5 ADHD rating scale* provides on the basis of 18 items two separate scales for symptoms of inattention and hyperactivity/impulsivity following DSM‐5 [American Psychiatric Association, 2013] criteria for ADHD, with higher scores indicating greater ADHD‐related problems. Psychiatric symptoms of anxiety and depression were measured using the *Development and Well‐Being Assessment* (DAWBA; Goodman et al., 2000), a semi‐structured parent/carer interview designed to generate prediction scores for ICD‐10 [World Health Organization, 1992] and DSM‐IV‐TR [American Psychiatric Association, 2000] psychiatric diagnoses. DAWBA scores reflect six levels of predication (i.e., from ~0.1% to >70%) of the probability of meeting clinically relevant diagnostic criteria for a disorder, ranging from very unlikely to probably. To facilitate comparisons and following Angold et al. [2012], we created a pooled anxiety prediction score reflecting an individual's highest risk score across a group of anxiety disorders (OCD, generalized anxiety, panic disorder, agoraphobia, PTSD, separation anxiety, social phobia, and specific phobia). For depression, the DAWBA generates a prediction score for major depression according to DSM‐IV and ICD‐10 criteria.

### *Data Analysis*

Statistical analyses were conducted using STATA software 15.0 [StataCorp, 2017]. Linear mixed effects models were used to test predictors of domain scores (Communication, Socialization, Daily Living) and ABC composite scores. A random effect for site was included to take into consideration the multilevel nature of the data and account for heterogeneity across sites. Estimates of total within‐site variance were obtained via the “mlt package” which computes *R* ^*2*^ values for multilevel models according to Snijders and Bosker [1994]. This affords to separately estimate the amount of variance explained at the within‐site (Level 1) and between‐site level (Level 2). The magnitude of an adaptive functioning deficit relative to intellectual ability was determined by calculating for each individual FSIQ‐adaptive functioning difference scores separately for VABS Domain (Socialization, Communication, Daily Living Skills) and Composite Scores (ABC). Participants with ASD were split into two groups depending on whether the magnitude of their FSIQ‐VABS ABC discrepancy (FSIQ‐VABS ABC) exceeded 15 standard score points (i.e., at least 1 *SD*), which according to the VABS manual [Sparrow et al., 2005], indicates a marked and clinically important discrepancy between an individual's cognitive ability and overall adaptive functioning skills. Analyses on IQ‐adaptive discrepancy scores were restricted to individuals who had an FSIQ‐VABS ABC impairment as defined above, which included 71% of participants of the total sample (263 of 369). These participants displayed on average a FSIQ‐VABS ABC difference score that exceeded two *SD*s (*M* = 35.41, 95%CI [33.8; 37.0], *SD* = 13.19; Range = 15–78). An analysis on the full sample (i.e., *N* = 369), that is, including those without clinically significant discrepancies between IQ and adaptive behavior, can be found in Supplementary Table 3. Multivariate Multiple Regression (MMR) was conducted on IQ‐adaptive discrepancy scores to take into account the possible inter‐dependency among VABS outcome variables. To account for mathematical coupling between FSIQ and discrepancy scores, correlation coefficients were adjusted using Oldham's method [Oldham, 1962] and regression coefficients are reported for illustrative purposes only. To assess the effect of FSIQ on FSIQ‐VABS discrepancy scores in this sub‐sample, a separate non‐mathematically‐coupled MMR on VABS scores was conducted and included the linear and quadratic effect of FSIQ, as well as all covariates. This allowed assessing whether the linear effect of FSIQ depends on FSIQ itself (i.e., the quadratic effect) and gives accurate estimates of the function relating FSIQ and VABS. The same set of predictors were included for both linear mixed effects models and MMR: age, sex, FSIQ, ASD symptomatology (ADOS CSS Social Affect and CSS RRB, SRS‐2, RBS‐R, SSP) and symptoms of associated psychiatric conditions (ADHD inattentiveness and hyperactivity/impulsivity, anxiety, depression). To increase confidence in the robustness of the results obtained, an α‐level of <0.01 was applied for all statistical analyses.

## Results

**Figure 1:**
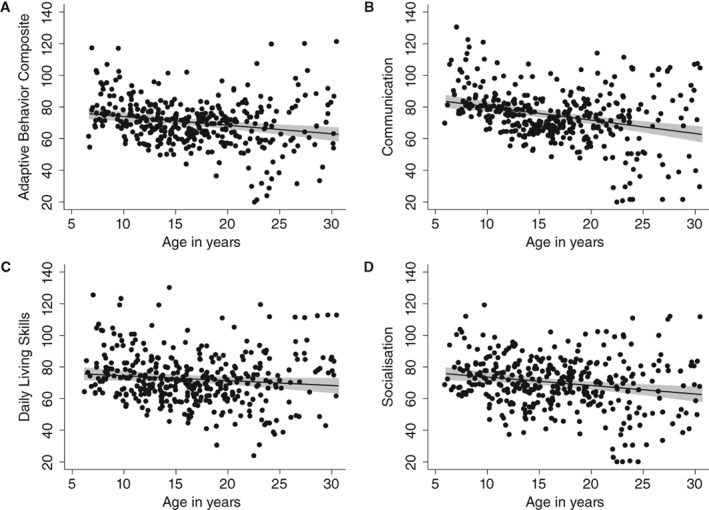
VABS standardized scores (age‐normalized reference value: M = 100, *SD* = 15) as a function of age. Points represent individual observations; linear regression line including 95% Confidence Interval.

Individuals with ASD demonstrated substantial impairments in adaptive behavior across all domains on standardized VABS scores (age‐normed reference value: *M* = 100, *SD* = 15). The most impaired adaptive behavior domain was Socialization, followed by Daily Living and the least impaired domain being Communication (Table 1; see Supplementary Materials for additional analyses and information on statistics and effect sizes). While there was great variability in VABS scores, domain and composite scores generally showed a pattern of greater adaptive functioning deficits with age (see Fig. 1). This was also reflected in significant but weak negative correlations between age and VABS domains (Socialization, Communication) and ABC scores (Supporting Information Table S1; *r* from −0.19 to −0.29, all *P*'s < 0.001). Splitting individuals with ASD and without ID across three age groups (children, adolescents and adults), children (Age 6 to 11 years: *M* = 77.98, 95% CI [75.19;80.76], *SD* = 12.98) had on average higher scores than adolescents (Age 12 to 17 years: *M* = 68.76, 95% CI [67.11;70.41], *SD* = 9.83) and adults (Age > 18 years: *M* = 67.17, 95% CI [64.23;70.11], *SD* = 18.00). Site differences for adaptive functioning ranged from minimal for the Socialization and Communication (Intra‐class correlation coefficient (ICC) = 0.14 and 0.06 respectively) to moderate for the Daily Living domain (ICC = 0.22) and ABC (ICC = 0.10).

**Figure 2:**
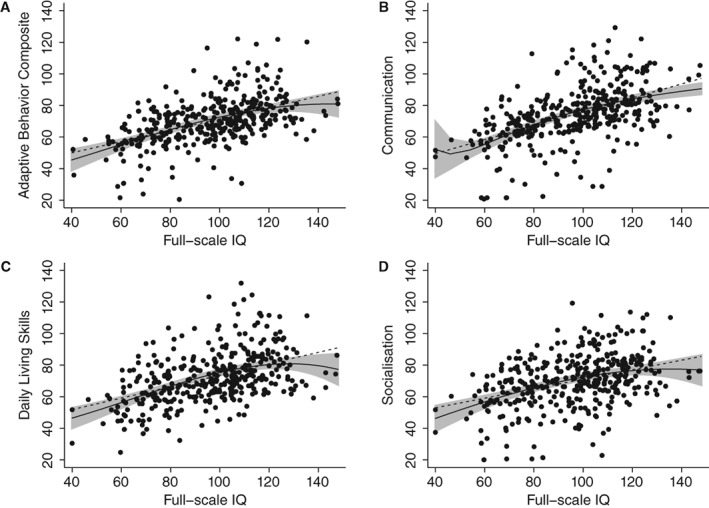
VABS standardized scores (age‐normalized reference value: M = 100, *SD* = 15) as a function of Full‐scale IQ. Points represent individual observations; Overlaid linear regression line (dotted line) and polynomial regression line (solid line) including 95% Confidence Interval.

Full‐scale IQ and VABS scores showed a significant moderate positive correlation, with higher IQ scores being associated with higher levels of adaptive functioning (Supporting Information Table S1; *r* from 0.38 to 0.53, all *P*'s < 0.0001). There was also some evidence of a curvilinear association (Fig. 2), such that the positive relationship between IQ and adaptive behavior becomes shallower at higher IQ levels. While this was particularly the case for the Daily Living and Socialization domain, it was least pronounced for the Communication domain, where the relationship between IQ and adaptive behavior appeared to follow a linear trend more closely. To test for this statistically, MMR models included both linear and quadratic terms for FSIQ and run for all VABS scores.

Testing all four equations simultaneously, the results revealed that the joint significance of the quadratic terms approached significance (*F*_(4,366)_ = 3.01, *P* = 0.018). Further exploratory individual comparisons for each VABS score and adopting a multiple comparison adjusted α‐level revealed that the quadratic term was only significant for Daily Living (*P* = 0.008), but not the other domains (all *P*'s > 0.054).

### *Predictors of Adaptive Functioning*

Results for linear mixed effects models are summarized in Table 2. Across VABS scores, the overall model was significant (Wald *x* ^2^_(17)_ > 130.57, *P* < 0.0001), with the proportion of variance accounted for ranging from 36% to 46% for VABS domains and 45% for the ABC score. With the exception of the daily living skills domain, age was a significant predictor across VABS ABC and domain‐level scores (all *P*'s < 0.001), with the expected negative association (i.e., lower VABS scores with older age, see Fig. 1). There was also a significant effect of FSIQ on VABS domain and composite scores (all *P*'s < 0.005), with higher IQ being associated with better adaptive functioning (see Fig. 2). There were no significant sex differences in adaptive behavior. For ASD symptom measures, higher SRS‐2 raw scores were associated with greater adaptive functioning deficits across domain and composite scores (all *P*'s < 0.002). No associations with adaptive functioning were found for ASD symptoms based on observation (ADOS), as well as restricted and repetitive behaviors (RBS‐R) and sensory symptoms (SSP). Similarly, psychiatric symptom measures were not significantly associated with VABS domain or ABC composite scores.

**Table 2:** Linear mixed effects models for VABS domain scores and Adaptive Behavior Composite

	Socialization			Daily living			Communication			Adaptive Behavior Composite		
Variable	*b SE (b)*	*z*	95% *CI*	*b SE (b)*	*z*	95% *CI*	*b SE (b)*	*z*	95% *CI*	*b SE (b)*	*z*	95% *CI*
Age	−1.13	−5.96*	[−1.50,‐0.76]	−0.38	−1.99	[−0.76,‐0.01]	−1.18	−6.60*	[−1.53,‐0.83]	−0.96	−6.10*	[−1.27,‐0.65]
	(0.19)			(0.19)			(0.18)			(0.16)		
Sex^a^		0.88			1.36			4.16			2.00	
												
FSIQ	0.14	2.80*	[0.04,0.23]	0.27	5.54*	[0.17,0.36]	0.30	6.32*	[0.21,0.39]	0.23	5.63*	[0.15,0.31]
	(0.05)			(0.05)			(0.05)			(0.04)		
ADOS CSS‐SA	0.33	0.86	[−0.43,1.09]	0.32	0.84	[−0.43,1.08]	−0.10	−0.26	[−0.82,0.63]	0.19	0.60	[−0.44,0.82]
	(0.39)			(0.39)			(0.37)			(0.32)		
ADOS CSS‐RRB	−0.04	−0.11	[−0.71,0.64]	−0.22	−0.63	[−0.89,0.46]	−0.02	−0.05	[−0.66,0.63]	−0.13	−0.45	[−0.69,0.43]
	(0.34)			(0.34)			(0.33)			(0.28)		
SRS‐2	−0.24	−5.38*	[−0.33,‐0.15]	−0.14	−3.03*	[−0.23,‐0.05]	−0.14	−3.19*	[−0.23,‐0.05]	−0.17	−4.59*	[−0.24,‐0.10]
	(0.05)			(0.05)			(0.04)			(0.04)		
RBS‐R	−0.15	−1.46	[−0.35,0.05]	−0.03	−0.26	[−0.23,0.18]	0.03	0.34	[−0.16,0.23]	−0.04	−0.49	[−0.21,0.13]
	(0.10)			(0.10)			(0.10)			(0.09)		
SSP	0.04	0.83	[−0.06,0.15]	0.05	0.91	[−0.05,0.15]	0.01	0.30	[−0.08,0.11]	0.04	0.93	[−0.04,0.13]
	(0.05)			(0.05)			(0.05)			(0.04)		
ADHD—Inattentiveness	0.68	1.74	[−0.09,1.45]	−0.22	−0.57	[−0.98,0.54]	−0.83	−2.20	[−1.57,‐0.09]	−0.17	−0.52	[−0.80,0.47]
	(0.39)			(0.39)			(0.38)			(0.32)		
ADHD—Hyper/Impul.	−0.46	−1.08	[−1.30,0.38]	0.21	0.50	[−0.62,1.05]	0.18	0.43	[−0.63,0.98]	0.03	0.09	[−0.66,0.72]
	(0.43)			(0.43)			(0.41)			(0.35)		
DAWBA—Depression	−1.72	−0.81	[−5.90,2.45]	−1.32	−0.62	[−5.49,2.84]	−3.58	−1.74	[−7.60,0.44]	−2.33	−1.32	[−5.77,1.12]
	(2.13)			(2.12)			(2.05)			(1.76)		
DAWBA—Anxiety	0.44	0.14	[−5.85,6.74]	2.12	0.66	[−4.15,8.38]	2.47	0.80	[−3.61,8.55]	1.95	0.74	[−3.24,7.14]
	(3.21)			(3.20)			(3.10)			(2.65)		
Bosker/Snijders *R* ^*2*^	0.443			0.356			0.456			0.449		
ICC^b^	0.14			0.22*			0.06			0.19*		

*Note. b*, regression coefficient, *SE(b)*, standard error of regression coefficient, *z*, z‐statistic, 95% *CI*, 95% Confidence Interval of regression coefficient; *R* ^*2*^, R‐squared estimate; FSIQ, Full‐scale IQ; ADOS CSS‐SA, RRB, autism diagnostic observation schedule calibrated severity scores for social affect and restricted and repetitive behaviors; SRS‐2, social responsiveness scale‐2; RBS‐R, repetitive behavior scale‐revised; SSP, short sensory profile; ADHD, DSM‐5 ADHD rating scale; DAWBA, development and well‐being assessment

a ANOVA Main effect (F‐statistic).

b ICC, Intra‐class correlation coefficient (ratio of between‐site variance to total variance).

* *P* < 0.01.

### *Discrepancy between Intellectual Ability and Adaptive Functioning*

**Figure 3:**
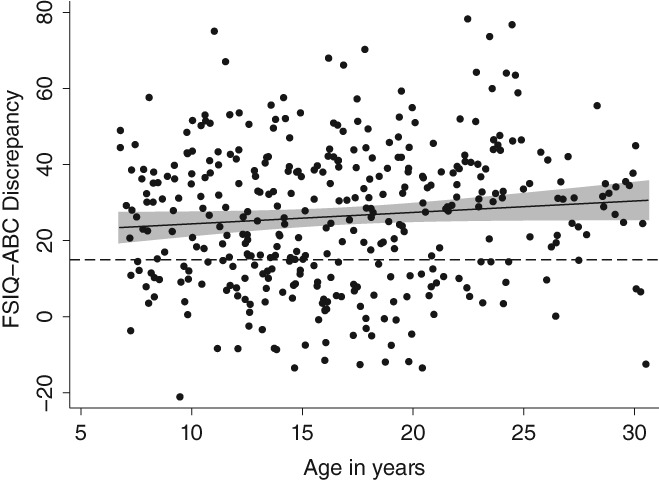
Distribution of FSIQ‐VABS ABC discrepancy scores by age.

Figure 3 highlights the heterogeneity in the magnitude of FSIQ‐VABS ABC discrepancy scores as a function of age in the sample. On the MMR, there were significant effects of age (all *P*'s < 0.001) across VABS domain and composite scores (with the exception of age for FSIQ‐Daily Living discrepancy scores; Table 3), with older age predicting larger discrepancy scores.

**Table 3:** Multivariate Multiple Regression models for FSIQ‐VABS Discrepancy Scores for Individuals with a FSIQ‐VABS ABC Discrepancy

	FSIQ‐Socialization			FSIQ‐Daily living			FSIQ‐Communication			FSIQ‐ABC		
Variable	*b SE (b)*	*t*	95% *CI*	*b SE (b)*	*t*	95% *CI*	*b SE (b)*	*t*	95% *CI*	*b SE (b)*	*t*	95% *CI*
Age	1.07	4.79*	[0.63,1.51]	0.11	0.47	[−0.34,0.56]	1.04	4.80*	[0.61,1.46]	0.79	4.68*	[0.46,1.13]
	(0.22)			(0.23)			(0.22)			(0.17)		
Sex^a^		0.28			0.25			2.89			1.21	
												
FSIQ^b^	0.72	–	–	0.64	–	–	0.55	–	–	0.65	–	–
	(0.08)			(0.08)			(0.08)			(0.06)		
ADOS CSS‐SA	−0.35	−0.78	[−1.25,0.55]	−0.46	−1.00	[−1.38,0.45]	−0.05	−0.11	[−0.92,0.82]	−0.33	−0.95	[−1.01,0.35]
	(0.45)			(0.46)			(0.44)			(0.34)		
ADOS CSS‐RRB	−0.21	−0.49	[−1.06,0.64]	0.84	1.92	[−0.03,1.70]	−0.35	−0.84	[−1.17,0.48]	0.11	0.33	[−0.54,0.76]
	(0.43)			(0.44)			(0.42)			(0.33)		
SRS‐2	0.26	4.61*	[0.15,0.36]	0.13	2.34	[0.02,0.24]	0.13	2.46	[0.03,0.24]	0.17	4.02*	[0.09,0.25]
	(0.06)			(0.06)			(0.05)			(0.04)		
RBS‐R	0.18	1.36	[−0.08,0.43]	0.10	0.74	[−0.16,0.35]	−0.01	−0.09	[−0.26,0.23]	0.08	0.77	[−0.12,0.27]
	(0.13)			(0.13)			(0.12)			(0.10)		
SSP	−0.08	−1.19	[−0.21,0.05]	−0.05	−0.79	[−0.18,0.08]	0.06	0.89	[−0.07,0.18]	−0.03	−0.51	[−0.12,0.07]
	(0.06)			(0.07)			(0.06)			(0.05)		
ADHD—Inattentiveness	−0.90	−1.87	[−1.85,0.05]	0.42	0.86	[−0.55,1.39]	0.11	0.23	[−0.81,1.03]	−0.06	−0.15	[−0.78,0.67]
	(0.48)			(0.49)			(0.46)			(0.36)		
ADHD—Hyper/Impul.	0.07	0.13	[−1.02,1.16]	−1.21	−2.16	[−2.31,‐0.10]	0.40	0.76	[−0.65,1.46]	−0.29	−0.70	[−1.12,0.53]
	(0.55)			(0.56)			(0.53)			(0.42)		
DAWBA—Depression	3.67	1.39	[−1.58,8.92]	−1.02	−0.38	[−6.35,4.31]	3.68	1.44	[−1.39,8.75]	2.20	1.09	[−1.78,6.18]
	(2.65)			(2.69)			(2.56)			(2.01)		
DAWBA—Anxiety	1.12	0.29	[−6.52,8.75]	−2.44	−0.62	[−10.19,5.31]	−1.71	−0.46	[−9.08,5.67]	−1.12	−0.38	[−6.91,4.67]
	(3.85)			(3.91)			(3.72)			(2.92)		

Note: *b*, regression coefficient, *SE(b)*, standard error of regression coefficient, *z*, z‐statistic, 95% *CI*, 95% Confidence Interval of regression coefficient; *R* ^*2*^, R‐squared estimate; FSIQ, Full‐scale IQ; ABC, adaptive behavior Composite; ADOS CSS‐SA, RRB, autism diagnostic observation schedule calibrated severity scores for social affect and restricted and repetitive behaviors; SRS‐2, social responsiveness scale‐2; RBS‐R, repetitive behavior scale‐revised; SSP, short sensory profile; ADHD, DSM‐5 ADHD rating scale; DAWBA, development and well‐being assessment.

ANOVA Main effect (F‐statistic).

Regression coefficients may reflect mathematical coupling and are therefore reported for illustrative purposes only.

* *P* < 0.01.

Age‐related differences in FSIQ‐VABS ABC discrepancy scores were however modest, with children (Age 6 to 11 years: *M* = 27.97, 95% CI [24.21;31.72], *SD* = 17.29) having on average lower discrepancy scores than adolescents (Age 12 to 17 years: *M* = 30.07, 95% CI [24.84;33.29], *SD* = 16.51), who in turn had lower discrepancy scores than adults (Age > 18 years: *M* = 33.34, 95% CI [30.31;36.37], *SD* = 16.40). Sex‐related comparisons were not significant. In relation to ASD symptom measures, higher SRS‐2 raw scores were significantly associated with greater discrepancy scores on the Socialization domain (*b* = 0.26, *P* < 0.001) and ABC (*b* = 0.17, *P* < 0.001), and was approaching significance for Daily Living Skills (*b* = 0.13, *P* = 0.021) and the Communication domain (*b* = 0.13, *P* = 0.015). There was no significant effect of RRB or sensory symptoms on FSIQ‐adaptive functioning discrepancy scores. Psychiatric symptom measures were also not significantly associated with FSIQ‐VABS discrepancy scores. When analyzing the full sample of participants, that is, including those with a FSIQ‐VABS ABC discrepancy of less than 15 standard score points, the same pattern of effects was observed (see Supporting Information Table S3). In line with the expected positive association between FSIQ and FSIQ‐VABS discrepancy scores (Supporting Information Table S2), the non‐mathematically‐coupled MMR on VABS scores revealed significant linear and quadratic effects of FSIQ on the Daily Living domain (*b*_*linear*_ = 2.13, *P* = 0.003; *b*_*quadratic*_ = −0.008, *P* = 0.013) and ABC (*b*_*linear*_ = 1.71, *P* = 0.001; *b*_*quadratic*_ = −0.006, *P* = 0.01) and was approaching significance on the socialization domain (*b*_*linear*_ = 1.52, *p* = 0.032; *b*_*quadratic*_ = −0.006, *P* = 0.078).

## Discussion

This study is the first to‐date to comprehensively assess how different aspects of the ASD phenotype and associated psychiatric symptoms are associated with adaptive behavior and ability‐adaptive discrepancies in ASD. The findings show that it is social‐communicative symptoms, but not sensory or repetitive symptoms or co‐occurring psychiatric symptoms including anxiety, depression and ADHD that are associated with lower adaptive functioning and greater ability‐adaptive function discrepancies. To further deconstruct these relationships at different levels of measurement (i.e., clinician‐rated, parent‐rated questionnaires) and across different domains of impairment, the ADOS, a well‐established diagnostic measure, was administered alongside a measure of autistic traits severity (SRS‐2) as well as specific measures of repetitive (RBS‐R) and sensory symptoms (SSP). A clear pattern of findings emerged, whereby more severe parent‐rated social symptoms (SRS‐2) were predictive of both lower adaptive functioning scores (across domain and composite scores) and greater IQ‐adaptive functioning discrepancy scores (Socialization domain and composite scores), while observer ratings of ASD symptoms (ADOS‐2) and parent‐rated sensory symptoms and RRB were not. Overall, these findings suggest that social aspects of the ASD phenotype, at least as captured by parent‐report trait measures, are critical factors in mediating the acquisition of adaptive competencies in real life situations. Social impairments across the lifespan may impede appropriate development of adaptive behavior skills by changing the experience of the environment and further restricting opportunities for learning in naturalistic settings. However, this does not imply that sensory symptoms and RRB, as well as symptoms of associated psychiatric conditions should be overlooked when designing interventions targeting adaptive skill acquisition. In fact, the correlational analysis highlighted small to moderate associations between sensory symptoms, RRB, associated psychiatric symptoms and adaptive functioning in ASD. When entered simultaneously in a regression model with other associated variables, these associations were however not significant. A recent longitudinal study also suggests that within individuals, the relationship between ASD symptoms and adaptive behavior over time is more complex than observed here, such that there exists only a small amount of “yoking” of developmental trajectories between these two constructs [Szatmari et al., 2015]. In other words, some individuals with more severe but stable ASD symptoms may show marked improvements in adaptive skills, reflecting their potential to acquire developmentally‐appropriate adaptive skills [Szatmari et al., 2015]. As the EU‐AIMS LEAP cohort will be followed into the future, we will be able to address a range of these complex interactions in more detail.

Of note is that SRS‐2 scores, based on parent‐report, showed the strongest association with Vineland scores, which were also parent‐reported. It is therefore possible that shared method variance, that is, the fact that the two measures share the same reporting method, may partially account for the strong relationship between SRS‐2 and Vineland scores. However, other parent‐report measures also included in the analysis to assess specific aspects of the ASD phenotype ‐restrictive and repetitive behaviors (RBS‐R) and sensory atypicalities (SSP) – had a reduced level of association with parent‐reported Vineland scores. This suggests that shared method variance between the SRS‐2 and Vineland is unlikely to fully account for the results observed. Another concern relates to the observation that the SRS‐2 taps into overlapping symptom constructs as the Vineland, and in particular social skills. While this is certainly the case for the Socialization domain, which shows the strongest association with SRS‐2 scores, SRS‐2 scores are also significantly associated with non‐social adaptive skills (e.g., Daily Living Skills). In fact, regression coefficients for SRS‐2 scores are equivalent for the Daily Living and Communication domain, suggesting a similar effect of ASD symptoms on adaptive behavior across different domains of “real‐world” functioning.

The results also replicate previous findings in several key areas, including the expected profile of impaired adaptive functioning in individuals with ASD with the largest impairments seen for Socialization, followed by Daily living skills and Communication; age‐related declines in adaptive functioning relative to age‐matched peers in a standardization sample; and a strong effect of IQ for adaptive behavior [Kanne et al., 2011; Pugliese et al., 2015]. Exploratory analyses also revealed a trend towards a curvilinear association between full‐scale IQ and adaptive functioning. This may indicate that IQ‐adaptive discrepancies become more pronounced with higher IQ, such that in high‐IQ individuals adaptive skills are less commensurate with cognitive abilities. Although these effects were modest and only significant at trend level, they are in line with another recent study [Chatham et al., 2018], suggesting that cognitive ability does not fully explain impairments in adaptive functioning in ASD, and particularly in higher‐ability individuals. It is also noteworthy that albeit significant due to the large sample studied, age had only a modest effect on adaptive scores.

Consistent with previous research [Klin et al., 2007; Kanne et al., 2011; Pugliese et al., 2015], most subjects (71% of the sample) demonstrated significant impairments in adaptive functioning relative to their IQ. Among those individuals with ASD with clinically significant ability‐adaptive discrepancies (i.e., a discrepancy score of at least 1 *SD*), the analysis revealed age‐ and IQ‐dependent effects, with older age and higher IQ being associated with larger FSIQ‐adaptive functioning discrepancy scores. Specifically, the analysis on VABS scores showed that while higher FSIQ is associated with higher VABS scores on the Daily Living domain and ABC (i.e., the linear effect of FSIQ), this relationship depends on FSIQ itself (i.e., it is lower at higher values; the quadratic effect). Overall, these findings suggest that the magnitude of deficits in real‐life skills relative to an individual's level of IQ was increasingly more pronounced in older compared to younger individuals as well as in those with higher cognitive abilities. Importantly, the analysis confirmed that these age‐ and IQ‐dependent effects uniquely contributed to variability in adaptive functioning.

There are limitations to the study. First, age‐related declines in adaptive functioning relative to age‐peers and a widening of an ability‐adaptive discrepancy through later childhood, adolescence and into adulthood may be accounted for by differences in services or interventions received, which was not investigated in the present analysis. This is a complex issue however, since interventions may differ in the onset, length, intensity, quality and type of intervention received, and any of these factors may have affected variability in adaptive functioning. Second, the cross‐sectional nature of the data leaves open possibilities for alternative interpretations. For example, it is not clear if the age‐related differences observed reflect true effects or are due to sampling differences across different ages. Third, the majority of participants in the LEAP sample had an elevated IQ compared to the total population of individuals with ASD, of whom around 50% have an intellectual disability [Charman, 2015; Lai, Lombardo, & Baron‐Cohen, 2014]. Thus, the findings need to be replicated in a larger sample of lower ability individuals with ASD. Although it is a notable limitation of the representativeness of the current sample that in common with many studies we excluded individuals with ASD with severe intellectual disability, it is rare for experimental biomarker studies to include participants with an IQ below 75. Finally, the current study reports on adaptive function drawn from the individual differences approach taken in conventional DSM/ICD psychiatric nosology but did not consider functioning within a wider biopsychosocial model. Recent work has adapted the WHO International Classification of Functioning Disability and Health to develop “core sets” specifically for ASD [Bölte et al., 2018]. We also did not consider patient‐nominated outcomes such as quality of life [van Heijst & Geurts, 2015; Oakley et al., 2018].

## Conclusion

Despite many individuals with ASD scoring well on standardized IQ tests and the expectation that this may translate into achieving positive outcomes, many individuals with ASD have difficulty coping in everyday life and are not able to fully capitalize upon their cognitive strengths to develop adaptive skills in real‐world contexts. Thus, the term ‘high functioning autism,’ sometimes used to refer to individuals with ASD without an intellectual disability (ID) is an inaccurate clinical descriptor when based solely on IQ, as adaptive functioning in the real‐world can be considerably impaired even for the most ‘high functioning’ individual. Given that outcome, particularly for higher‐ability individuals, is more related to adaptive behavior skills than cognitive factors [Farley et al., 2009], identifying the impediments to adaptive skills acquisition has important clinical implications for interventions. According to the present findings, core social communication ASD symptoms seem to play a larger role in contributing to variability in adaptive functioning and ability‐adaptive discrepancies in ASD than sensory and repetitive ASD symptoms and co‐occurring psychiatric symptoms. The results suggest that the severity of ASD social communication symptoms constitutes a barrier to the development of more sophisticated adaptive functioning skills, respectively they depend on both, social communication abilities and intellectual functioning. Interventions targeting adaptive skills acquisition should therefore be tailored according to developmental stage, level of cognitive ability and take account of ASD symptom severity, in particular social‐communicative abilities to better support individuals with ASD to live independently and navigate the social world. The strengths of the study include the large sample size, the broad range of ages and different levels of cognitive functioning studied, and the use of multi‐method, multi‐informant instruments that capture *different* aspects of the ASD phenotype, as well a range of commonly associated psychiatric symptoms (ADHD, anxiety, depression). This extends previous studies significantly by comprehensively assessing adaptive functioning in a well‐powered, well‐characterized sample of individuals with ASD.

## Conflict of interests

Tobias Banaschewski has served in an advisory or consultancy role for Actelion, Hexal Pharma, Lilly, Lundbeck, Medice, Neurim Pharmaceuticals, Novartis, and Shire. He received conference support or speaker's fee by Lilly, Medice, Novartis and Shire. He is/has been involved in clinical trials conducted by Shire and Viforpharma. He received royalties from Hogrefe, Kohlhammer, CIP Medien, and Oxford University Press. The present work is unrelated to the above grants and relationships. Sven Bölte receives royalties for the German and Swedish KONTAKT manuals, and adaptations of the ADI‐R, ADOS, and SRS from Hogrefe Publishers. Sven Bölte has in the last 3 years acted as an author, consultant or lecturer for Shire, Medice, Roche, Eli Lilly, Prima Psychiatry, GLGroup, System Analytic, Kompetento, Expo Medica, Prophase, and receives royalties for text books and diagnostic tolls from Huber/Hogrefe, Kohlhammer and UTB. Jan Buitelaar has been in the past 3 years a consultant to/member of advisory board of/and/or speaker for Janssen Cilag BV, Eli Lilly, Lundbeck, Shire, Roche, Novartis, Medice and Servier. He is not an employee of any of these companies, and not a stock shareholder of any of these companies. He has no other financial or material support, including expert testimony, patents, and royalties.

Chris Chatham, Lindsay Ham and Will Spooren are employees at F. Hoffmann‐La Roche Ltd. Emily Simonoff receives support from the National Institute of Health Research (NIHR) Programme Grant for Applied Research, a Senior Investigator Award and though the NIHR South London and Maudsley NIHR Biomedical Research Centre. She also receives funding from the European Medicine Innovative Medicines Initiative (EU‐AIMS), the Medical Research Council, The Economic and Social Research Council, Autistica and the Maudsley Charity. No other conflict of interests declared.

## Supporting information


**Appendix S1:** Supplementary Materials

**Table S1.** Correlations between VABS adaptive behavior domain and standard scores, age, IQ, and clinical measures
**Table S2.** Correlations between FSIQ‐VABS discrepancy scores age, IQ, and clinical measures
**Table S3.** Multivariate Multiple Regression models for FSIQ‐VABS discrepancy scores in the whole sample

## References

[aur2081-bib-0001] American Psychiatric Association . (2000). Diagnostic and Statistical Manual of Mental Disorders (4th ed.). Washington, DC: American Psychiatric Association.

[aur2081-bib-0002] American Psychiatric Association . (2013). Diagnostic and Statistical Manual of Mental Disorders (5th ed.). Arlington, VA: Author.

[aur2081-bib-0003] Angold, A. , Erkanli, A. , Copeland, W. , Goodman, R. , Fisher, P. W. , & Costello, E. J. (2012). Psychiatric diagnostic interviews for children and adolescents: a comparative study. Journal of the American Academy of Child & Adolescent Psychiatry, 51(5), 506–517. 22525957 10.1016/j.jaac.2012.02.020 PMC3336098

[aur2081-bib-0004] Ashwood, K. L. , Tye, C. , Azadi, B. , Cartwright, S. , Asherson, P. , & Bolton, P. (2015). Brief report: Adaptive functioning in children with ASD, ADHD and ASD+ ADHD. Journal of Autism and Developmental Disorders, 45(7), 2235–2242. 25614019 10.1007/s10803-014-2352-y

[aur2081-bib-0005] Bal, V. H. , Kim, S.‐H. , Cheong, D. , & Lord, C. (2015). Daily living skills in individuals with autism spectrum disorder from 2 to 21 years of age. Autism, 19(7), 774–784. 25922445 10.1177/1362361315575840 PMC4912002

[aur2081-bib-0006] Bodfish, J. W. , Symons, F. J. , Parker, D. E. , & Lewis, M. H. (2000). Varieties of Repetitive Behavior in Autism: Comparisons to Mental Retardation. Journal of Autism and Developmental Disorders, 30(3), 237–243. 11055459 10.1023/a:1005596502855

[aur2081-bib-0007] Bölte, S. , Mahdi, S. , de Vries, P. J. , Granlund, M. , Robison, J. E. , Shulman, C. , … Zwaigenbaum, L. (2018). The Gestalt of functioning in autism spectrum disorder: Results of the international conference to develop final consensus International Classification of Functioning, Disability and Health core sets. Autism, 1, 1362361318755522. 10.1177/1362361318755522 PMC6376609 29378422

[aur2081-bib-0008] Bölte, S. , & Poustka, F. (2002). The relation between general cognitive level and adaptive behavior domains in individuals with autism with and without co‐morbid mental retardation. Child Psychiatry and Human Development, 33(2), 165–172. 12462353 10.1023/a:1020734325815

[aur2081-bib-0009] Charman, T. (2015). Variability in Neuro‐Developmental Disorders Evidence from Autism Spectrum Disorders In Van Herwegen J. & Riby D. M. (Eds.), Neurodevelopmental Disorders : Research Challenges and Solutions. Hove, U.K: Psychology Press.

[aur2081-bib-0010] Charman, T. , Loth, E. , Tillmann, J. , Crawley, D. , Wooldridge, C. , Goyard, D. , … Buitelaar, J. K. (2017). The EU‐AIMS Longitudinal European Autism Project (LEAP): clinical characterisation. Molecular Autism, 8(1), 27. 28649313 10.1186/s13229-017-0145-9 PMC5481972

[aur2081-bib-0011] Charman, T. , Pickles, A. , Simonoff, E. , Chandler, S. , Loucas, T. , & Baird, G. (2011). IQ in children with autism spectrum disorders: data from the Special Needs and Autism Project (SNAP). Psychological Medicine, 41(3), 619–627. 21272389 10.1017/S0033291710000991

[aur2081-bib-0012] Chatham, C. H. , Taylor, K. I. , Charman, T. , Liogier D'ardhuy, X. , Eule, E. , Fedele, A. , … Bolognani, F. (2018). Adaptive behavior in autism: Minimal clinically important differences on the Vineland‐II. Autism Research, 11(2), 270–283. 28941213 10.1002/aur.1874 PMC5997920

[aur2081-bib-0013] Constantino, J. N. , & Gruber, C. P. (2012). Social Responsiveness Scale (2nd ed.). Los Angeles, CA: Western Psychological Services.

[aur2081-bib-0014] De Bildt, A. , Sytema, S. , Kraijer, D. , Sparrow, S. , & Minderaa, R. (2005). Adaptive functioning and behaviour problems in relation to level of education in children and adolescents with intellectual disability. Journal of Intellectual Disability Research, 49(9), 672–681. 16108984 10.1111/j.1365-2788.2005.00711.x

[aur2081-bib-0015] Duncan, A. W. , & Bishop, S. L. (2015). Understanding the gap between cognitive abilities and daily living skills in adolescents with autism spectrum disorders with average intelligence. Autism, 19(1), 64–72. 24275020 10.1177/1362361313510068 PMC7398153

[aur2081-bib-0016] Farley, M. A. , McMahon, W. M. , Fombonne, E. , Jenson, W. R. , Miller, J. , Gardner, M. , … Ritvo, R. A. (2009). Twenty‐year outcome for individuals with autism and average or near‐average cognitive abilities. Autism Research, 2(2), 109–118. 19455645 10.1002/aur.69

[aur2081-bib-0017] Farmer, C. , Swineford, L. , Swedo, S. E. , & Thurm, A. (2018). Classifying and characterizing the development of adaptive behavior in a naturalistic longitudinal study of young children with autism. Journal of Neurodevelopmental Disorders, 10(1), 1. 29329511 10.1186/s11689-017-9222-9 PMC5795287

[aur2081-bib-0049] Goodman, R. , Ford, T. , Richards, H. , Gatward, R. , & Meltzer, H. (2000). The Development and Well‐Being Assessment: description and initial validation of an integrated assessment of child and adolescent psychopathology. Journal of Child Psychology and Psychiatry, 41(5), 645–655. 10946756

[aur2081-bib-0018] Kanne, S. M. , Gerber, A. J. , Quirmbach, L. M. , Sparrow, S. S. , Cicchetti, D. V. , & Saulnier, C. A. (2011). The role of adaptive behavior in autism spectrum disorders: Implications for functional outcome. Journal of Autism and Developmental Disorders, 41(8), 1007–1018. 21042872 10.1007/s10803-010-1126-4

[aur2081-bib-0019] Klin, A. , Saulnier, C. A. , Sparrow, S. S. , Cicchetti, D. V. , Volkmar, F. R. , & Lord, C. (2007). Social and communication abilities and disabilities in higher functioning individuals with autism spectrum disorders: The Vineland and the ADOS. Journal of Autism and Developmental Disorders, 37(4), 748–759. 17146708 10.1007/s10803-006-0229-4

[aur2081-bib-0020] Kraper, C. K. , Kenworthy, L. , Popal, H. , Martin, A. , & Wallace, G. L. (2017). The Gap Between Adaptive Behavior and Intelligence in Autism Persists into Young Adulthood and is Linked to Psychiatric Co‐morbidities. Journal of Autism and Developmental Disorders, 47(10), 3007–3017.2871053210.1007/s10803-017-3213-2

[aur2081-bib-0021] Lai, M.‐C. , Lombardo, M. V. , & Baron‐Cohen, S. (2014). Autism. The Lancet, 383(9920), 896–910. 10.1016/S0140-6736(13)61539-1 24074734

[aur2081-bib-0022] Lord, C. , Risi, S. , Lambrecht, L. , Cook, E. H. , Leventhal, B. L. , DiLavore, P. C. , … Rutter, M. (2000). The autism diagnostic observation schedule—Generic: A standard measure of social and communication deficits associated with the spectrum of autism. Journal of Autism and Developmental Disorders, 30(3), 205–223. 11055457

[aur2081-bib-0023] Lord, C. , Rutter, M. , DiLavore, P. C. , Risi, S. , Gotham, K. , & Bishop, S. (2012). Autism Diagnostic Observation Schedule, Second Edition (ADOS‐2) Manual (Part I): Modules 1–4. Torrance, CA: Western Psychological Services.

[aur2081-bib-0024] Loth, E. , Charman, T. , Mason, L. , Tillmann, J. , Jones, E. J. H. , Wooldridge, C. , … Buitelaar, J. K. (2017). The EU‐AIMS Longitudinal European Autism Project (LEAP): design and methodologies to identify and validate stratification biomarkers for autism spectrum disorders. Molecular Autism, 8(24), 1–19. 28649312 10.1186/s13229-017-0146-8 PMC5481887

[aur2081-bib-0025] Mouga, S. , Almeida, J. , Café, C. , Duque, F. , & Oliveira, G. (2015). Adaptive profiles in autism and other neurodevelopmental disorders. Journal of Autism and Developmental Disorders, 45(4), 1001–1012. 25241010 10.1007/s10803-014-2256-x

[aur2081-bib-0026] Oakley, B. , Tillmann, J. , Ahmad, J. , Crawley, D. , Caceres, A. S. J. , Holt, R. J. , . . . Loth, E. (2018). What drives variation in quality of life for individuals on the autism spectrum? The impact of core symptoms, anxiety and depression. *Manuscript in preparation* .

[aur2081-bib-0027] Oldham, P. (1962). A note on the analysis of repeated measurements of the same subjects. Journal of Chronic Diseases, 15(10), 969–977. 13939936 10.1016/0021-9681(62)90116-9

[aur2081-bib-0028] Paul, R. , Miles, S. , Cicchetti, D. , Sparrow, S. , Klin, A. , Volkmar, F. , … Booker, S. (2004). Adaptive behavior in autism and pervasive developmental disorder‐not otherwise specified: Microanalysis of scores on the Vineland adaptive behavior scales. Journal of Autism and Developmental Disorders, 34(2), 223–228. 15162940 10.1023/b:jadd.0000022612.18116.46

[aur2081-bib-0029] Perry, A. , Flanagan, H. E. , Geier, J. D. , & Freeman, N. L. (2009). Brief report: The Vineland Adaptive Behavior Scales in young children with autism spectrum disorders at different cognitive levels. Journal of Autism and Developmental Disorders, 39(7), 1066–1078. 19234777 10.1007/s10803-009-0783-7 PMC2759870

[aur2081-bib-0030] Pugliese, C. E. , Anthony, L. , Strang, J. F. , Dudley, K. , Wallace, G. L. , & Kenworthy, L. (2015). Increasing adaptive behavior skill deficits from childhood to adolescence in autism spectrum disorder: Role of executive function. Journal of Autism and Developmental Disorders, 45(6), 1579–1587. 25398602 10.1007/s10803-014-2309-1 PMC4433442

[aur2081-bib-0031] Rutter, M. , Le Couteur, A. , & Lord, C. (2003). Autism Diagnostic Interview‐Revised. Los Angeles, CA: Western Psychological Services.

[aur2081-bib-0032] Smith, L. E. , Maenner, M. J. , & Seltzer, M. M. (2012). Developmental trajectories in adolescents and adults with autism: The case of daily living skills. Journal of the American Academy of Child & Adolescent Psychiatry, 51(6), 622–631. 22632621 10.1016/j.jaac.2012.03.001 PMC3361701

[aur2081-bib-0033] Snijders, T. A. , & Bosker, R. J. (1994). Modeled variance in two‐level models. Sociological Methods & Research, 22(3), 342–363.

[aur2081-bib-0034] Sparrow, S. S. , Balla, D. , & Cicchetti, D. V. (1984). Vineland Adpative Behavior Scales: Interview edition survey form manual. Circle Press, MN: American Guidance Service.

[aur2081-bib-0035] Sparrow, S. S. , Cicchetti, D. V. , & Balla, D. (2005). The Vineland Adaptive Behavior Scales (2nd ed.). Circle Pines, MN: American Guidance Service.

[aur2081-bib-0036] StataCorp . (2017). Stata Statistical Software: Release 15. College Station, TX: StataCorp LLC.

[aur2081-bib-0037] Szatmari, P. , Bryson, S. , Duku, E. , Vaccarella, L. , Zwaigenbaum, L. , Bennett, T. , & Boyle, M. H. (2009). Similar developmental trajectories in autism and Asperger syndrome: from early childhood to adolescence. Journal of Child Psychology and Psychiatry, 50(12), 1459–1467. 19686332 10.1111/j.1469-7610.2009.02123.x

[aur2081-bib-0038] Szatmari, P. , Georgiades, S. , Duku, E. , Bennett, T. A. , Bryson, S. , Fombonne, E. , … Vaillancourt, T. (2015). Developmental trajectories of symptom severity and adaptive functioning in an inception cohort of preschool children with autism spectrum disorder. JAMA Psychiatry, 72(3), 276–283. 25629657 10.1001/jamapsychiatry.2014.2463

[aur2081-bib-0039] Tomchek, S. D. , & Dunn, W. (2007). Sensory processing in children with and without autism: A comparative study using the short sensory profile. American Journal of Occupational Therapy, 61(2), 190–200. 17436841 10.5014/ajot.61.2.190

[aur2081-bib-0040] van Heijst, B. F. , & Geurts, H. M. (2015). Quality of life in autism across the lifespan: A meta‐analysis. Autism, 19(2), 158–167. 24443331 10.1177/1362361313517053

[aur2081-bib-0041] Wechsler, D. (1991). Wechsler Intelligence Scale for Children (3rd ed.). San Antonio, TX: Psychological Corporation.

[aur2081-bib-0042] Wechsler, D. (1997). Wechsler Adult Intelligence Scale (3rd ed.). San Antonio, TX: The Psychological Corporation.

[aur2081-bib-0043] Wechsler, D. (2003). Wechsler Intelligence Scale for Children (4th ed.). San Antonio, TX: Psychological Corporation.

[aur2081-bib-0044] Wechsler, D. (2008). Wechsler Adult Intelligence Scale–Fourth Edition. San Antonio, TX: Pearson.

[aur2081-bib-0045] Wechsler, D. (2011). Wechsler Abbreviated Scale of Intelligence–Second Edition (WASI‐II). San Antonio, TX: NCS Pearson.

[aur2081-bib-0046] World Health Organization . (1992). The ICD‐10 classification of mental and behavioural disorders: Clinical descriptions and diagnostic guidelines (Vol. 1). Geneva: World Health Organization.

