# Supplementary material for: Investigating the factors underlying adaptive functioning in autism in the EU‐AIMS Longitudinal European Autism Project
Source: Autism Res. 2019 Feb 11;12(4):645–57. doi: 10.1002/aur.2081 (PMC6519242; doi:10.1002/aur.2081)
Supplement: Supplementary file 1 — Appendix S1: Supplementary Materials [file AUR-12-645-s001.docx]

**Supplementary Materials**

*Measures - Autism Diagnostic Observation Schedule*

Depending on an individual’s language level and age, participants were administered one of several modules of the *Autism Diagnostic Observation Schedule* (ADOS-G; Lord et al., 2000; ADOS-2; Lord et al., 2012). Most participants received the ADOS-2 (Module 1: *N* = 1; Module 2: *N* = 3; Module 3: *N* = 153; Module 4: *N* = 218), while a minority of participants received the ADOS-G (Module 3: *N* = 8; Module 4: *N* = 26)*.* Item scores for ADOS-G were first converted to ADOS-2 algorithm scores, which were then used to derive a Calibrated Severity Score (CSS). CSS provide a standardised ASD severity measure for the core symptom domains of Social Communication (i.e. Social Affect), and Restricted and Repetitive Behaviours (RRB), as well as an overall indicator of ASD severity (CSS Total). CSS are based on a 10-point severity metric, with higher scores indicating more severe ASD symptoms.

In those instances where ADOS CSS could not be computed because of missing ADOS-G or ADOS-2 item score data (*N* = 19; 5% of total observations), ADOS CSS were imputed by using a multiple imputation procedure with chained equations in STATA (‘mi impute chained’ in Stata15, StataCorp, 2017). Variables included in the imputation procedure, due to their high correlation with ADOS CSS, were ADOS-G Communication and Social Interaction Total scores for ADOS CSS Social Affect (Module 3 & 4: *r* = .92) and ADOS-G Stereotyped Behaviours and Restricted Interests Total scores for ADOS CSS RRB (Module 3: *r* = .81; Module 4: *r* = .66) and run for Module 3 and 4 separately. To satisfy the missing at random (MAR) assumption, site was included as an auxiliary variable in the model and a total of 30 imputations were run for each outcome variable.

*Results - Characterisation of adaptive functioning*

Across all VABS domains, individuals with ASD showed significant deficits in adaptive functioning, with the most impaired domain being Socialisation, followed by Daily Living and the least impaired domain being Communication (Table 1). Paired sample *t*-tests adopting a multiple comparison adjusted α-level of 0.017 (i.e. 0.05/3 = 0.017) comparing each VABS domain were run. They confirmed that these were statistically significant differences of impairment, with the Socialisation domain being more impaired than Daily Living domain (*t*(372) = 3.54, *p* = .0005, d = .18) and Communication domain (*t*(373) = 5.89, *p* = .8.617e-09, d = .30). The Daily living domain was also more impaired than the Communication domain (*t*(372) = 3.03, *p* = .003, d = .16).

In addition, in individuals with ASD, all VABS domain and standard scores fell well below (i.e. around 2 *SD*s) the age-normed reference value of 100 (*SD* = 15; one-sample *t*-test: all *p*’s < 4.036e-96, Cohen’s *d* from 1.5 to 2.0), highlighting the magnitude of adaptive functioning impairment in this sample compared to typically developing age-matched peers. Most VABS adaptive behaviour domain and ABC standard scores showed significant but weak negative correlations with age (at *p* <.001), with largest correlations seen for the Communication domain (*r* = -.29*, p* <.0001; Supplementary Table 1), followed by ABC standard scores (*r* = -.21*, p* <.0001) and the Socialisation domain (*r* = -.19*, p* =.0002)*.*

- Supplementary Table 1 about here -

Since this study uses a cross-sectional design, any significant correlations with age could have be an artefact of IQ differences between the younger and the older participants (*N* = 343; FSIQ ≥ 75), which may be related to differences in recruitment patterns or developmental changes in IQ. To rule out this possibility, the sample was divided by a median age split (Median = 16.3 years) and an independent sample *t*-test was performed to test for FSIQ score differences between age groups. No significant IQ differences between age groups were observed (*M_Young_* = 102.2, *SD_Young_* = 15.2 vs. *M_Old_* = 104.4, *SD_Old_* = 15.5; *t*(337) = 1.30, *p* = .193, d = .14), confirming the robustness of the results above.

Overall, there were significant negative correlations between ASD symptom measures and VABS scores across most domain and standard scores ranging from weak to moderate (Supplementary Table 1; *r* from -.19 to -.55, all *p*’s < .001), with greater symptom severity associated with more impaired adaptive functioning. In contrast, for measures of associated psychiatric conditions of ADHD, anxiety and depression, the findings were more mixed. While ADHD symptoms of Inattentiveness and to a lesser degree Hyperactivity/Impulsivity showed significant negative correlations with VABS scores (*r* from -.18 to -.33, all *p*’s < .001), there were no significant associations between anxiety, depression and VABS standardised scores.

**Supplementary References**

Lord, C., Risi, S., Lambrecht, L., Cook, E. H., Leventhal, B. L., DiLavore, P. C., . . . Rutter, M. (2000). The Autism Diagnostic Observation Schedule—Generic: A Standard Measure of Social and Communication Deficits Associated with the Spectrum of Autism. *Journal of Autism and Developmental Disorders, 30(3),* 205-223.

Lord, C., Rutter, M., DiLavore, P. C., Risi, S., Gotham, K., & Bishop, S. (2012). *Autism Diagnostic Observation Schedule, Second Edition (ADOS-2) Manual (Part I): Modules 1–4*. Torrance, CA: Western Psychological Services.

Oldham, P. (1962). A note on the analysis of repeated measurements of the same subjects. *Journal of chronic diseases, 15(10),* 969-977.

StataCorp. (2017). *Stata Statistical Software: Release 15*. College Station, TX: StataCorp LLC.
