# Supplementary material for: Investigating the factors underlying adaptive functioning in autism in the EU‐AIMS Longitudinal European Autism Project
Source: Autism Res. 2019 Feb 11;12(4):645–57. doi: 10.1002/aur.2081 (PMC6519242; doi:10.1002/aur.2081)
Supplement: Supplementary file 2 — Table S1. Correlations between VABS adaptive behavior domain and standard scores, age, IQ, and clinical measures Table S2. Correlations between FSIQ‐VABS discrepancy scores age, IQ, and clinical measures Table S3. Multivariate Multiple Regression models for FSIQ‐VABS discrepancy scores in the whole sample [file AUR-12-645-s002.docx]

**Supplementary Table 1** Correlations between VABS adaptive behaviour domain and standard scores, age, IQ, and clinical measures

|  | VABS Socialisation | VABS Daily Living | VABS Communication | VABS ABC |
| --- | --- | --- | --- | --- |
| Age | -0.19^*^ | -0.11 | -0.29^*^ | -0.21^*^ |
| Full-scale IQ | 0.38^*^ | 0.46^*^ | 0.53^*^ | 0.50^*^ |
| ADOS CSS-SA | -0.19^*^ | -0.16 | -0.22^*^ | -0.19^*^ |
| ADOS CSS-RRB | -0.21^*^ | -0.24^*^ | -0.14 | -0.21^*^ |
| SRS-2 | -0.55^*^ | -0.47^*^ | -0.32^*^ | -0.49^*^ |
| RBS-R | -0.44^*^ | -0.36^*^ | -0.20^*^ | -0.36^*^ |
| SSP | 0.39^*^ | 0.38^*^ | 0.13 | 0.34^*^ |
| ADHD - Inattentiveness | -0.29^*^ | -0.33^*^ | -0.23^*^ | -0.31^*^ |
| ADHD - Hyperactivity/Impulsivity | -0.25^*^ | -0.18^*^ | -0.10 | -0.19^*^ |
| DAWBA - Anxiety | -0.10 | -0.07 | 0.03 | -0.04 |
| DAWBA - Depression | -0.07 | -0.00 | 0.01 | -0.02 |

Note: VABS ABC = Vineland-II Adaptive Behavior Composite; ADOS CSS-SA, RRB = Autism Diagnostic Observation Schedule Calibrated Severity Scores for Social Affect and Restricted and Repetitive Behaviours; SRS-2 = Social Responsiveness Scale – 2; RBS-R = Repetitive Behavior Scale – Revised; SSP = Short Sensory Profile; ADHD = DSM-5 ADHD rating scale; DAWBA = Development and Well-Being Assessment

* *p* <0.001

**Supplementary Table 2** Correlations between FSIQ-VABS discrepancy scores age, IQ, and clinical measures

|  | FSIQ-VABS Socialisation | FSIQ-VABS Daily Living | FSIQ-VABS Communication | FSIQ-VABS ABC |
| --- | --- | --- | --- | --- |
| Age | 0.13 | 0.05 | 0.23^*^ | 0.14 |
| FSIQ^†^ | 0.22 | 0.23 | 0.19 | 0.34 |
| ADOS CSS-SA | -0.02 | -0.08 | 0.01 | -0.06 |
| ADOS CSS-RRB | 0.08 | 0.11 | 0.01 | 0.07 |
| SRS-2 | 0.28^*^ | 0.23^*^ | 0.07 | 0.21^*^ |
| RBS-R | 0.28^*^ | 0.23^*^ | 0.05 | 0.20 |
| SSP | -0.27^*^ | -0.29^*^ | -0.05 | -0.24 |
| ADHD - Inattentiveness | 0.05 | 0.10 | -0.00 | 0.05 |
| ADHD - Hyperactivity/Impulsivity | 0.04 | -0.02 | -0.11 | -0.04 |
| DAWBA - Anxiety | 0.10 | 0.08 | 0.01 | 0.06 |
| DAWBA - Depression | 0.10 | 0.04 | 0.04 | 0.06 |

Note: VABS ABC = Vineland-II Adaptive Behavior Composite; ADOS CSS-SA, RRB = Autism Diagnostic Observation Schedule Calibrated Severity Scores for Social Affect and Restricted and Repetitive Behaviours; FSIQ = Full-scale IQ; SRS-2 = Social Responsiveness Scale – 2; RBS-R = Repetitive Behavior Scale – Revised; SSP = Short Sensory Profile; ADHD = DSM-5 ADHD rating scale; DAWBA = Development and Well-Being Assessment
†Correlation coefficients adjusted following Oldham’s method: $Corr\left[ x-y,\frac{x+y}{2} \right]=\frac{s_{x}^{2}-s_{y}^{2}}{\sqrt{(s_{x}^{2}+s_{y}^{2})^{2}-4r_{xy}^{2}}s_{x}^{2}s_{y}^{2}}$ , where $s_{x}^{2}$ = Variance of FSIQ, $s_{y}^{2}$ = Variance of VABS score, $r_{xy}$ = Correlation
between FSIQ and VABS score (see Supplementary Table 1); * p <0.001

**Supplementary Table 3** Multivariate Multiple Regression models for FSIQ-VABS discrepancy scores in the whole sample

|  | FSIQ-Socialisation | | |  | FSIQ-Daily living | | |  | FSIQ-Communication | | |  | FSIQ-ABC | | |
| --- | --- | --- | --- | --- | --- | --- | --- | --- | --- | --- | --- | --- | --- | --- | --- |
| Variable | *b*  *SE (b)* | *t* | 95% *CI* |  | *b*  *SE (b)* | *t* | 95% *CI* |  | *b*  *SE (b)* | *t* | 95% *CI* |  | *b*  *SE (b)* | *t* | 95% *CI* |
| Age | 1.13 | 5.78^*^ | [0.75,1.52] |  | 0.50 | 2.42 | [0.09,0.91] |  | 1.14 | 6.14^*^ | [0.77,1.51] |  | 0.94 | 5.76^*^ | [0.62,1.26] |
|  | (0.20) |  |  |  | (0.21) |  |  |  | (0.19) |  |  |  | (0.16) |  |  |
| Sex^†^ |  | 0.73 |  |  |  | 1.01 |  |  |  | 3.30 |  |  |  | 1.58 |  |
|  |  |  |  |  |  |  |  |  |  |  |  |  |  |  |  |
| FSIQ^‡^ | 0.88 | - | - |  | 0.76 | - | - |  | 0.71 | - | - |  | 0.79 | - | - |
|  | (0.05) |  |  |  | (0.06) |  |  |  | (0.05) |  |  |  | (0.04) |  |  |
| ADOS CSS-SA | -0.43 | -1.06 | [-1.24,0.38] |  | -0.49 | -1.14 | [-1.33,0.36] |  | 0.22 | 0.57 | [-0.54,0.98] |  | -0.25 | -0.74 | [-0.92,0.42] |
|  | (0.41) |  |  |  | (0.43) |  |  |  | (0.39) |  |  |  | (0.34) |  |  |
| ADOS CSS-RRB | 0.43 | 1.25 | [-0.25,1.12] |  | 0.97 | 2.66 | [0.25,1.69] |  | -0.04 | -0.12 | [-0.69,0.61] |  | 0.47 | 1.63 | [-0.10,1.04] |
|  | (0.35) |  |  |  | (0.36) |  |  |  | (0.33) |  |  |  | (0.29) |  |  |
| SRS-2 | 0.25 | 5.02^*^ | [0.15,0.35] |  | 0.14 | 2.78^*^ | [0.04,0.25] |  | 0.14 | 2.95^*^ | [0.05,0.23] |  | 0.18 | 4.26^*^ | [0.09,0.26] |
|  | (0.05) |  |  |  | (0.05) |  |  |  | (0.05) |  |  |  | (0.04) |  |  |
| RBS-R | 0.19 | 1.71 | [-0.03,0.41] |  | 0.12 | 1.01 | [-0.11,0.35] |  | -0.04 | -0.35 | [-0.24,0.17] |  | 0.07 | 0.80 | [-0.11,0.26] |
|  | (0.11) |  |  |  | (0.12) |  |  |  | (0.11) |  |  |  | (0.09) |  |  |
| SSP | -0.07 | -1.29 | [-0.18,0.04] |  | -0.10 | -1.64 | [-0.21,0.02] |  | -0.01 | -0.13 | [-0.11,0.10] |  | -0.06 | -1.34 | [-0.16,0.03] |
|  | (0.06) |  |  |  | (0.06) |  |  |  | (0.05) |  |  |  | (0.05) |  |  |
| ADHD - Inattentiveness | -0.51 | -1.20 | [-1.34,0.33] |  | 0.49 | 1.09 | [-0.39,1.36] |  | 0.77 | 1.93 | [-0.02,1.56] |  | 0.31 | 0.87 | [-0.39,1.00] |
|  | (0.42) |  |  |  | (0.44) |  |  |  | (0.40) |  |  |  | (0.35) |  |  |
| ADHD – Hyper/Impul. | 0.07 | 0.15 | [-0.83,0.96] |  | -0.82 | -1.72 | [-1.76,0.12] |  | -0.17 | -0.40 | [-1.02,0.67] |  | -0.40 | -1.06 | [-1.14,0.35] |
|  | (0.45) |  |  |  | (0.48) |  |  |  | (0.43) |  |  |  | (0.38) |  |  |
| DAWBA – Depression | 1.44 | 0.63 | [-3.10,5.98] |  | 0.82 | 0.34 | [-3.95,5.59] |  | 3.35 | 1.54 | [-0.95,7.64] |  | 2.05 | 1.07 | [-1.73,5.83] |
|  | (2.30) |  |  |  | (2.42) |  |  |  | (2.17) |  |  |  | (1.92) |  |  |
| DAWBA – Anxiety | -0.84 | -0.24 | [-7.74,6.05] |  | -2.57 | -0.70 | [-9.81,4.68] |  | -1.96 | -0.59 | [-8.48,4.57] |  | -2.11 | -0.73 | [-7.86,3.63] |
|  | (3.49) |  |  |  | (3.67) |  |  |  | (3.30) |  |  |  | (2.91) |  |  |

Note: *b* = regression coefficient, *SE(b)* = Standard Error of regression coefficient, *z* = z-statistic, 95% *CI* = 95% Confidence Interval of regression coefficient; *R^2^* = unadjusted R-squared estimate; FSIQ = Full-scale IQ; ABC = Adaptive Behavior Composite; ADOS CSS-SA, RRB = Autism Diagnostic Observation Schedule Calibrated Severity Scores for Social Affect and Restricted and Repetitive Behaviours; SRS-2 = Social Responsiveness Scale – 2; RBS-R = Repetitive Behavior Scale – Revised; SSP = Short Sensory Profile; ADHD = DSM-5 ADHD rating scale; DAWBA = Development and Well-Being Assessment; * *p* <.01
^†^ANOVA Main effect (F-statistic); ^‡^Regression coefficients may reflect mathematical coupling and are therefore reported for illustrative purposes only
